# Cognitive Dissonance–Based Priming Intervention: Randomized Encouragement With in-the-Wild Phishing Simulation Attack in Health Care

**DOI:** 10.2196/68051

**Published:** 2026-06-01

**Authors:** Prosper Kandabongee Yeng, Muhammad Ali Fauzi, Arnstein Vestad, Bian Yang, Katrien De Moor, Christian Jacobsen, John-Bosco Diekuu, Meriem Bettayeb

**Affiliations:** 1 Department of Computer Science and IT, College of Engineering, Abu Dhabi University, Al Ain, Abu Dhabi, United Arab Emirates, +971 0506991627; 2 Department of Informatics Engineering, Faculty of Computer Science, University of Brawijaya, Malang, Indonesia; 3 Department of Information Security and Communication Technology, Faculty of Information Technology and Electrical Engineering, Norwegian University of Science and Technology (NTNU), Gjøvik, Norway; 4 Department of Information Security and Communication Technology, Faculty of Information Technology and Electrical Engineering, Norwegian University of Science and Technology (NTNU), Trondheim, Norway; 5 Department of Security and Risk Governance, Aidn AS, Oslo, Norway; 6 Department of Machine Learning and Computer Vision, School of Computing, Engineering and Technology, Robert Gordon University, Aberdeen, United Kingdom

**Keywords:** cognitive dissonance, phishing simulation, health care, randomized encouragement, psychological incentive, health belief model, protection motivation theory, cognition, phishing, phishing attacks, cybersecurity, phishing attempts, health care staff, security, Norway

## Abstract

**Background:**

Phishing remains a dominant initial attack vector in health care, exploiting psychological factors such as urgency and authority. Despite extensive investment in technical controls and awareness training, health care staff remain highly susceptible in real operational conditions. Cognitive dissonance (CD), the discomfort arising from inconsistencies between beliefs and actions, has been proposed as a mechanism to disrupt unsafe rationalization at the moment of exposure, but has rarely been evaluated in live organizational settings using objective behavioral outcomes.

**Objective:**

This study examined whether a brief CD-based priming intervention, delivered immediately prior to a real-world phishing simulation, was associated with differences in phishing susceptibility among health care staff. Secondary objectives explored whether CD exposure was associated with directional differences in security-related perceptions and self-reported practices.

**Methods:**

A 2-stage hybrid randomized-encouragement experiment was conducted at a large Norwegian hospital. In Stage 1, staff were randomly assigned to a control or CD-primed condition and completed a survey assessing security perceptions and self-reported practices (n=62). In Stage 2, an in-the-wild phishing simulation was sent to all staff, enabling objective measurement of phishing susceptibility via observed link-click behavior. Behavioral outcomes were analyzed across 3 groups—control (n=34), CD-primed (n=32), and neutral nonresponders (n=753)—using a prespecified omnibus chi-square test as the sole confirmatory analysis. Survey-based multivariate and univariate analyses were treated as exploratory due to limited sample size and variable construct reliability.

**Results:**

Due to voluntary uptake, only a subset of randomized participants received the intervention. Observed phishing click rates were 65% (22/34) in the control group, 44% (14/32) in the CD-primed group, and 53% (396/753) in the neutral group. The omnibus chi-square test did not detect a statistically significant association between group membership and click behavior (*χ*²_2_=3.00; n=819; *P*=.22; Cramér V=0.06). Descriptive comparisons within the randomized subset suggested lower click rates in the CD-primed group, but effect estimates were imprecise and associated with wide CIs. Survey-based analyses indicated group differences across combined psychological constructs; however, several constructs exhibited low internal consistency, and follow-up analyses were underpowered.

**Conclusions:**

In a real-world hospital phishing simulation, pre-exposure CD priming was associated with a directional but statistically nonsignificant pattern of reduced phishing click behavior. This evidence does not establish a reliable behavioral effect, and construct-level findings are exploratory. CD-based prompts may serve as a lightweight behavioral signal in real-world conditions, but larger, fully randomized, and longitudinal studies with improved psychometric validation are needed before such interventions can be considered reliable complements to established cybersecurity controls.

## Introduction

### Background

Phishing remains the dominant initial attack vector in health care, accounting for a substantial proportion of breaches between 2022 and 2025 through credential theft, business email compromise, and ransomware deployment [1-3]. Despite significant investment in email filtering and endpoint defenses, health care employees continue to be exposed to highly contextualized phishing campaigns that exploit clinical workflows, trust relationships, professional hierarchies, and time pressure [2-6]. Consequently, health care has become one of the costliest sectors for data breaches, with human-driven social engineering outweighing purely technical exploitation [2,2,4,7-9]. These attacks not only compromise data confidentiality but also disrupt care delivery, delay clinical workflows, and threaten patient safety [9-11].

To explain persistent phishing susceptibility, prior research has increasingly applied psychological frameworks such as the Health Belief Model (HBM) and Protection Motivation Theory (PMT) [7,10,12,13]. These models examine how perceived severity (PS), perceived vulnerability (PV), self-efficacy (SE), and response efficacy (RE) influence individual security behavior. However, most health care phishing studies rely on self-reported intentions, survey-based perceptions, or post-hoc evaluations conducted in training contexts rather than during real operational attacks [4-6,14-16]. As a result, existing interventions rarely disrupt unsafe cognition at the moment of threat exposure, nor do they validate effectiveness using objective behavioral outcomes [3,4,11,14].

The objectives of this study are therefore outlined as follows:

- Delivering the first cognitive dissonance (CD)–based prephishing intervention tested in a live hospital;
- Experimentally linking CD to shifts in HBM or PMT perceptions;
- Measuring real click behavior during an in-the-wild phishing event;
- Demonstrating a theory-driven, low-cost behavioral security control with ecological validity.

### CD-Based in-the-Wild Phishing Intervention

CD theory explains how individuals experience psychological discomfort when their beliefs conflict with their actions, such as recognizing phishing risks while still clicking urgent or authoritative emails [17-19]. To reduce this discomfort, individuals often rationalize risky behavior through neutralization techniques, including denial of harm, denial of responsibility, or appeals to higher organizational goals [11,20-22]. In health care contexts, such rationalizations have been observed in email-policy violations, unsafe password practices, and delayed incident reporting justified by clinical urgency [4,10,10,23].

Phishing attackers deliberately exploit these same psychological mechanisms by embedding urgency, authority, fear, and trust cues into realistic clinical narratives, thereby intensifying cognitive conflict and impairing reflective judgment [3,6,23,24]. Recent reviews emphasize that such human-factor vulnerabilities remain inadequately addressed by purely technical controls and awareness-only training approaches [8,9,13,25].

This study introduces a CD-based priming intervention delivered immediately before an in-the-wild phishing simulation at one of Norway’s largest hospitals. Unlike traditional training, deterrence-based sanctions, or postincident awareness programs [11,13-15,25], CD priming operates as a proactive, preattack behavioral control that directly targets rationalization before exposure. The intervention is lightweight, theory-driven, and embedded within routine organizational communication, making it suitable for real clinical environments characterized by time pressure and high cognitive load [3,3,9].

Methodologically, the study advances prior work in 3 ways. First, it deploys CD priming immediately prior to a live operational phishing event rather than within simulated, laboratory, or training-only contexts [5,11,14,26]. Second, it integrates CD with established HBM and PMT constructs to examine shifts in theory-driven security perceptions [7,10,12,13,15]. Third, it validates intervention effectiveness using objective behavioral outcomes—actual link-click behavior—while minimizing ethical risk by avoiding credential harvesting and excessive deception [27,28]. To our knowledge, no prior health care study has combined CD-based priming, health-behavior theory, and in-the-wild behavioral measurement within a single experimental design [3,9,29].

### Hypothesis Formulation and Contributions

The primary objective of this study was to examine whether exposure to a CD-based priming intervention was associated with differences in observed phishing susceptibility during a real-world phishing simulation (hypothesis 1). Secondary analyses explored whether CD exposure was associated with directional differences in psychological security perceptions and self-reported security practices.

The following hypothesis and research questions were therefore specified:

#### Hypothesis 1 (Confirmatory)

Exposure to a CD-based priming intervention is associated with differences in observed phishing susceptibility, measured by link-click behavior during an in-the-wild phishing simulation.

#### Research Question (RQ) 1 (Exploratory)

Is exposure to a CD-based priming message associated with directional differences in selected security-related perceptions derived from the Health Belief Model (HBM) and Protection Motivation Theory (PMT), including PV, PS, SE, RE, perceived barriers (PBs), and cues to action (CA)?

#### RQ2 (Exploratory)

Is exposure to a CD-based priming message associated with directional differences in self-reported security practices related to password management, incident reporting, email handling, and mobile-device security?

## Methods

### Overview

This study used a 2-stage randomized encouragement design [30,31], involving approximately 830 health care staff who were randomly assigned at the invitation stage to receive either a control or a CD-primed questionnaire [27,32,33]. Only a subset of invited staff completed the questionnaire and were therefore exposed to the intervention (control: n=42; CD-primed: n=40), while nonresponse was treated as nonreceipt rather than exclusion, consistent with established methodological guidance [34]. Randomization, therefore, applies only to encouragement and survey exposure, not to subsequent receipt of the phishing email or behavioral outcome measurement.

**Figure 1.**
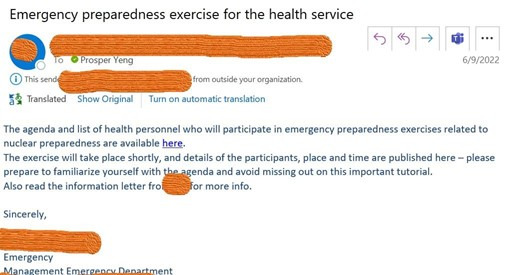
Phishing attack simulation message.

In Stage 2, an in-the-wild phishing simulation [4] message (as shown in Figure 1) was sent to all staff with valid email addresses, enabling objective measurement of phishing click behavior under real-world conditions. Primary outcome analyses for the randomized component were conducted among questionnaire respondents who received valid phishing emails. These participants were the only ones plausibly exposed to both the intervention and the behavioral outcome. In parallel, a large Neutral group comprising staff who did not complete the questionnaire but received the in-the-wild phishing email was included as an observational comparison. Analyses involving this group are reported descriptively and interpreted without causal inference in accordance with STROBE (Strengthening the Reporting of Observational Studies in Epidemiology) guidelines [35], while the randomized encouragement component is reported following CONSORT (Consolidated Standards of Reporting Trials) principles [36], ensuring transparent reporting of participant flow, uptake, and inferential boundaries.

**Figure 2.**
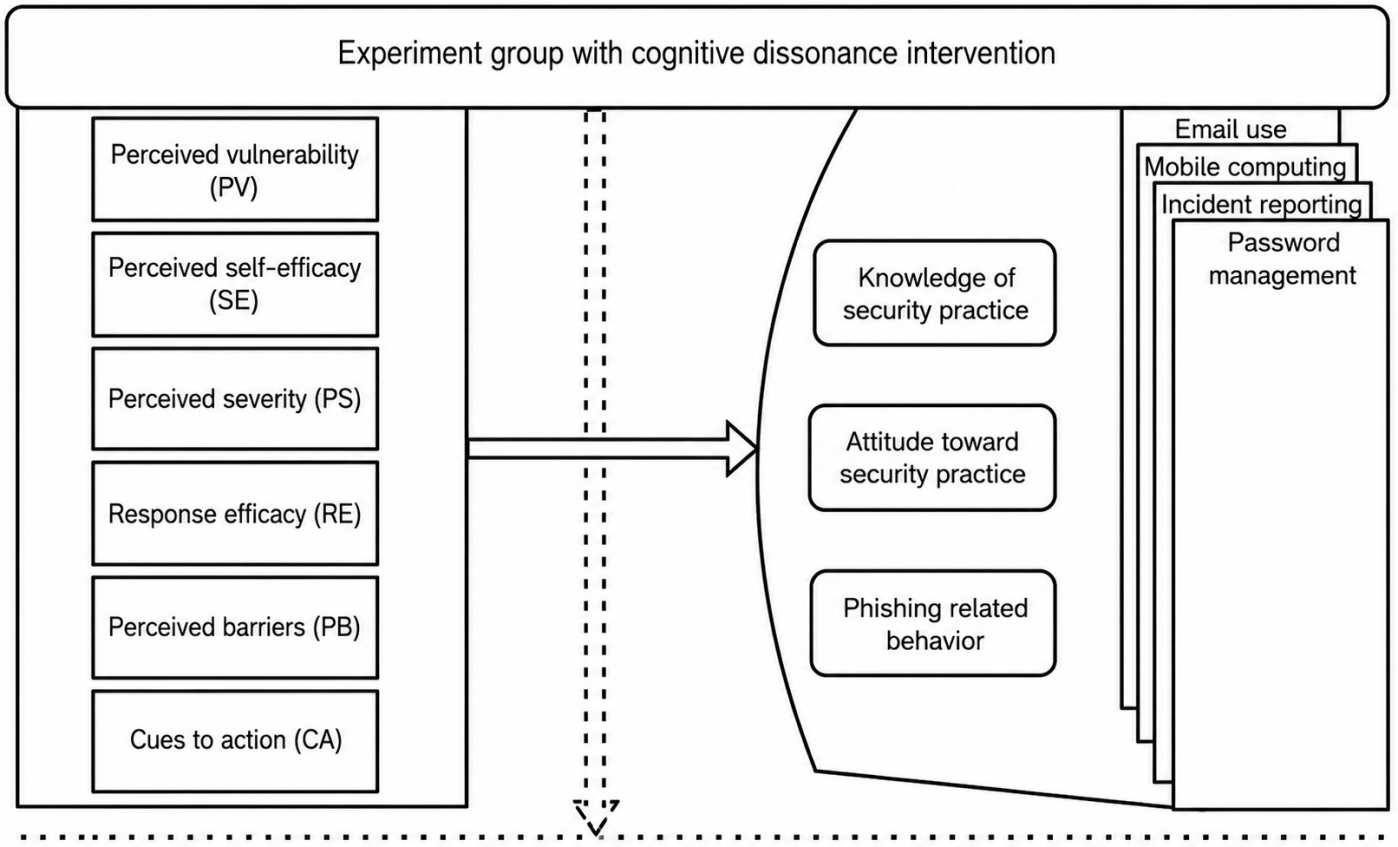
Experiment model for psychological incentive.

Behavioral outcomes were assessed independently of survey participation. Valid delivery and outcome records were available for 819 participants, who formed the sample used for behavioral analysis. These participants fell into 3 groups based on their prior exposure to the CD message:

- Control group: survey respondents who did not receive the CD message and had valid phishing-outcome data (n=34).
- Experiment group: survey respondents who received the CD message and had valid phishing-outcome data (n=32). The architecture is shown in Figure 2.
- Neutral group: all remaining staff who did not receive any CD-related priming but successfully received the phishing email (n=753).

The difference between the invited population (~830) and the Stage 2 analyzed sample (819) reflects staff with undeliverable emails. This grouping structure enabled the study to (1) evaluate the causal effect of the CD intervention on psychological and self-reported outcomes among survey respondents and (2) compare their actual phishing susceptibility against a large neutral group not exposed to any experimental priming.

As an incentive, participants in both stages were offered a free lunch at the hospital canteen and also entered into a draw in which 10 individuals could win a US $50 gift card [37,38].

### Behavioral Outcome: Phishing Click Susceptibility

Actual behavioral phishing susceptibility was measured by recording objective link-click behavior during the in-the-wild phishing simulation and coding it as a binary outcome (clicked vs not clicked) across 3 groups, including control (n=34), CD-primed (n=32), and neutral (n=753).

The primary, prespecified analysis was an omnibus Pearson chi-square test of independence (3×2 contingency table; 2-sided *α*=.05). While this test was designated a priori as the primary behavioral analysis, causal inference is limited to the randomized comparison between the control and CD-primed groups; comparisons involving the neutral group are observational.

Effect size was quantified using Cohen w and Cramér V, and group-specific click rates were reported with 95% CIs (Wilson method) [39]. Pairwise comparisons between the CD-primed and control groups were reported descriptively using risk difference, relative risk, and odds ratio with 95% CIs and treated as exploratory due to limited sample size [40].

A post hoc power assessment for the omnibus test was reported descriptively to characterize statistical sensitivity, rather than to support inferential claims. All analyses were conducted using the statsmodels Python (Python Software Foundation) library [16,41].

### Ethical, Privacy, and Security Measures With an in-the-Wild Study

In this experiment, a questionnaire and an in-the-wild study were combined. The hybrid approach was essential, as the survey provided a basis for the researchers to understand participants’ intended phishing security behavior, while the in-the-wild study tested participants’ actual phishing security practice (ie, the clicking action). An in-the-wild study is a type of phishing simulation in which the researcher conducts a phishing attack on participants in their natural working environment without prior warning to observe real-world security behavior under realistic conditions. Unlike laboratory-based or survey-based studies, in-the-wild studies capture authentic user responses but raise additional ethical and methodological considerations [4,27].

### Survey Instrument

**Figure 3.**
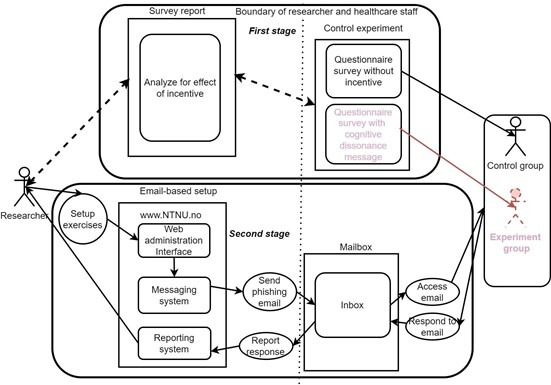
Experiment setup.

**Figure 4.**
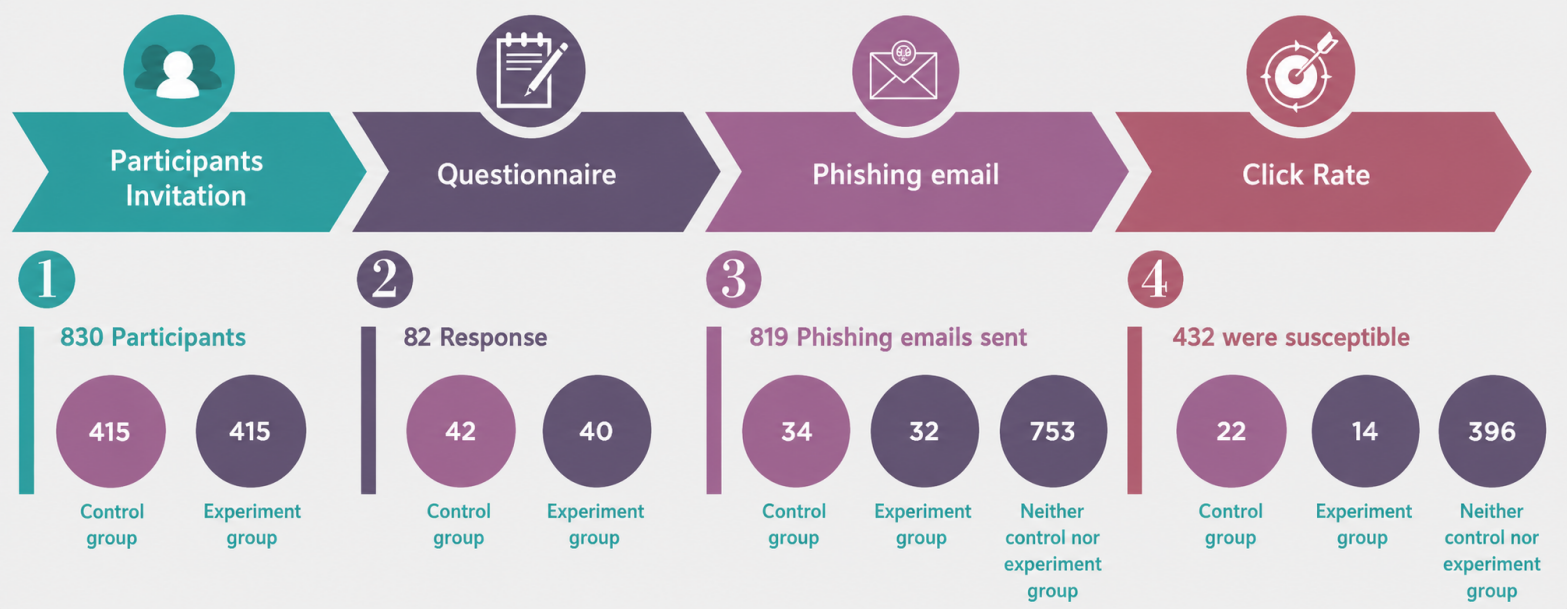
Response rate.

**Figure 5.**
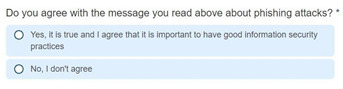
Cognitive dissonance message agreement for experiment group.

In the first section of the questionnaire, we adopted the approach of Parsons et al [42], the human aspect of the information security questionnaire. This consists of 42 items that measure knowledge, attitude, and behavior (KAB) regarding phishing, as well as risk factors associated with password management, email use, incident reporting, and mobile device use. A 5-point Likert scale was used in this instrument. These areas of security practice are considered more vulnerable to phishing attacks. Additionally, approximately 25 items in this instrument measured other psychological constructs, including PV, SE, CA, PB, and RE. Figure 2 shows the questionnaire structure. The instrument was developed with an online, secure Norwegian version of a survey system called Nettskjema [43]. The questionnaire was then divided into 2 groups, a control group and the experiment group. The difference between them was that the experiment group questionnaire included a CD message, as shown in Multimedia Appendix 1, and in the model in Figure 3. Participants also indicated in the questionnaire if they believe in the CD message as shown in Figure 4. The questionnaire for the control group did not include the CD message item. The instrument was then pretested with 4 PhD students and a professor specializing in cybersecurity. Issues, including complex terminologies and the length of the CD message, were identified and resolved. Measures were also taken against error variances [44]. Error variance can be caused by preexisting factors that introduce differences among the study groups, beyond the treatment effect. In this regard, the health care personnel were randomly assigned to reduce potential biases. Additionally, the participants were asked not to share their questionnaires with others. Attention checkers [45] were among the survey items in the study, and these required the participants to choose specified responses. Participants who fail to correctly answer 2 of the 3 attention checkers are inferred to have not paid attention while answering the questionnaire. As a result, 2 records were discarded. This has been one of the most popular methods for improving survey response quality without compromising research findings. The participants also had to either agree or disagree with the cognitive dissonance message, as shown in Figure 5.

### Phishing Simulation Setup and Experiment Process

A phishing simulation tool, known as Gophish (Jordan Wright) [28], was set up on a server, as illustrated in Figure 3. It has a feature that allows an attacker to simulate a phishing email and send it to a target. It can also record click events on links in phishing emails.

During the initial setup, the simulated phishing email was tested with the staff of the target hospital; however, it was flagged as spam by the service provider’s email filtering system. Since the study goal was not to test the technical email security controls of the target facility, we collaborated with the providers who configured the email system to allow the phishing simulation email to land in the inboxes of the targeted participants. The email message content is shown in Multimedia Appendix 2.

The in-the-wild phishing simulation was implemented using the open-source GoPhish platform, which was configured to deliver a single simulated phishing email to staff with valid institutional email addresses [28]. The platform automatically recorded email delivery status and objective link-click events, which were used as the primary behavioral outcome measure [11,26,29,46-50].

**Figure 6.**
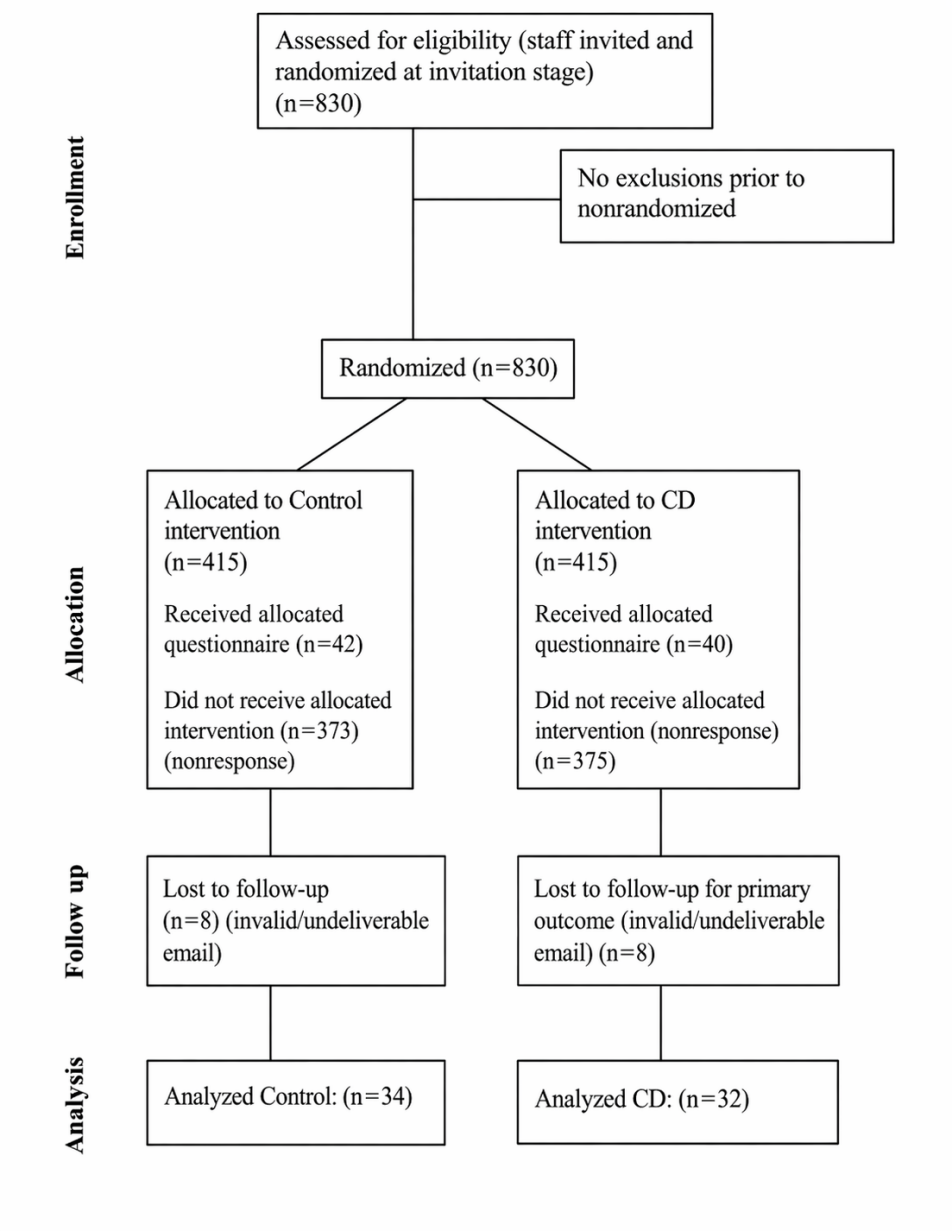
CONSORT (Consolidated Standards of Reporting Trials) diagram.

The flow of participants in both the controlled experiment and the observational study is shown in Figures6  7, respectively. The CONSORT and STROBE checklists have also been provided in Checklist 1 and Checklist 2, respectively.

**Figure 7.**
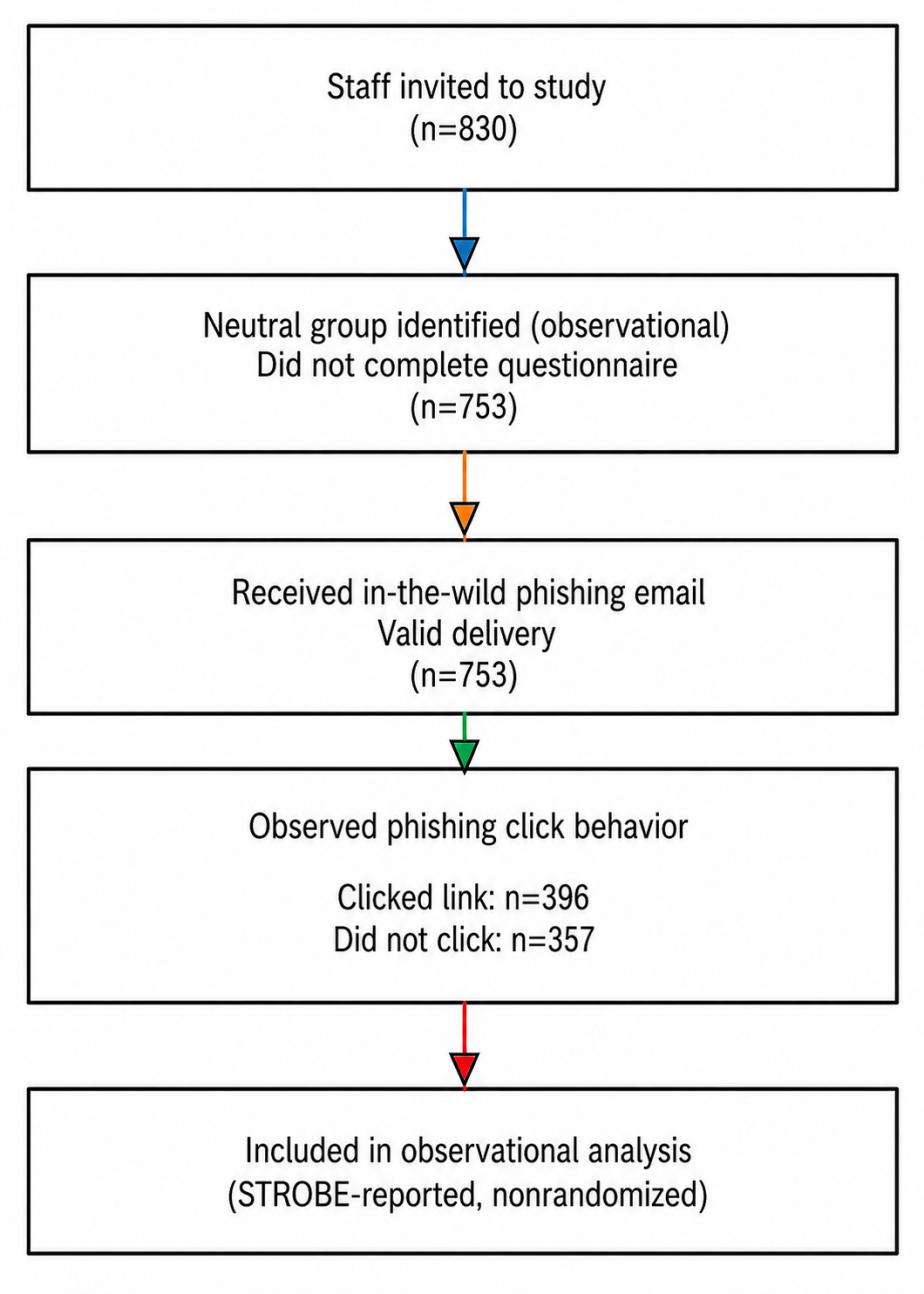
STROBE (Strengthening the Reporting of Observational Studies in Epidemiology) diagram.

Fourteen dependent scale variables representing psychosocial constructs derived from the Health Belief Model and Protection Motivation Theory were included in the exploratory multivariate analysis [51,52]. Additionally, a minimum of one independent variable is required with at least 2 categorical groups. Our study also meets this condition with an independent 2-level design: in the first stage, there are 2 levels (control and experiment), and in the second stage, there are 3 groups (neutral, control, and experiment). A multivariate analysis of variance (MANOVA) assumes multivariate normality. The test also requires homogeneity of covariate metrics, and the dependent variables must not be multicollinear. A sufficient sample size is also needed in MANOVA. A sample size is required for each level of the independent variable. The rule of thumb requires 30 participants per group level [53]. The actual behavior (AB) variable has the lowest number of participants in the control and experimental groups, 30 and 32, respectively, indicating that the study met the minimum requirement [54]. Cronbach α was used in this study because it is a widely used measure of reliability [40].

### Ethical Considerations

Prior to the launch of the phishing study, a press release was issued to administrators. Additionally, during the launch of the attack, data protection and the well-being of the participants were considered by providing a debriefing and obtaining informed consent [27]. Having followed these measures, this study obtained ethical clearance from the targeted hospital, the Regional Committees for Medical and Health Research Ethics of Norway (REK), and the Norwegian Center for Research Data (NSD). A phishing simulation was then conducted via email in this experiment.

## Results

### Overview

To ensure methodological transparency and inferential clarity, the analytical strategy was explicitly aligned with the study objectives. The omnibus chi-square test of independence comparing click behavior across the control, CD-primed, and neutral groups (n=819) was designated a priori as the sole confirmatory test of behavioral impact (hypothesis 1). All survey-based multivariate and univariate analyses, conducted on a substantially smaller subsample (n=62), were treated as exploratory and hypothesis-generating due to limited statistical power and variable construct reliability. This alignment ensures that confirmatory claims are restricted to adequately powered, objective behavioral outcomes, while construct-level findings are interpreted conservatively and used to inform future research design. This section presents the analysis of the results, covering descriptive statistics, click rates, reliability, normality tests, and the significance of the studies.

### Descriptive Statistics

All participants in the CD condition agreed with the presented message. As shown in Table 1, a total of 80 records from the survey were analyzed out of 82 participants. Two records were removed from the analysis because they did not pass the attention checks that were placed in the questionnaire [45].

**Table 1.** Descriptive statistics of demographic variables (N=80).

Variable category		n (%)	
Sex			
Male		58 (72.5)	
Female		22 (27.5)	
Age range (years)			
21‐30		14 (17.5)	
31‐40		23 (28.8)	
41‐50		18 (22.5)	
51‐60		16 (20.0)	
*>*60		9 (11.3)	
Position			
Administrator		6 (7.5)	
Nurse		40 (50.0)	
Doctor		31 (38.8)	
Others		3 (3.8)	
Years of experience			
1‐5		9 (11.3)	
6‐10		19 (23.8)	
11‐15		10 (12.5)	
16‐20		13 (16.3)	
*>*20		29 (36.3)	
Knowledge of phishing attacks			
No knowledge		12 (15.0)	
Basic		23 (28.8)	
Medium		26 (32.5)	
High		15 (18.8)	
Very high		3 (3.8)	
Professional		1 (1.3)	
Opinion in cognitive dissonance			
Not available		40 (50.0)	
Agree		38 (95.0)	
Disagree		2 (5.0)	

Furthermore, among these participants, 72.5% (58/80) were males, while 27.5% (22/80) were females. The age group 31-40 years old had the highest proportion (23/80, 28%), while participants >60 years old had the lowest proportion (9/80, 11.3%). Regarding the participants, primarily doctors, nurses, and administrators participated. Nurses comprised more than half of the total participants (40/80, 50%), followed by doctors (31/80, 38%) and administrators (6/80, 7.5%). In terms of years of work experience, participants with >20 years of experience were comparatively more numerous (29/80, 36.3%), as shown in Table 1. Meanwhile, the participants also shared their phishing security practice knowledge. Most of them (26/80, 32.5%) had medium knowledge, 28% (23/80) had basic knowledge, and 15% (12/80) reported no knowledge of phishing security practices. Additionally, participants in the experiment group shared their opinion on the treatment effect of CD. Out of the 40 participants in the experiment group, 38 (95%) agreed with the effectiveness of the treatment measure. To control for these confounding variables, the randomization approach [55] was used to assign participants to the 2 groups in this study.

As shown in Table 2, descriptive statistics were computed for all dependent variables across study groups. Overall, mean values for security practice measures were generally higher in the control group than in the experimental group. Box’s test of equality of covariance matrices was conducted to assess the assumption that the covariance matrices of the dependent variables were equivalent across groups. The test yielded a Box’s mean value of 153.081 with a nonsignificant *P* value (*P*=.24), indicating no evidence of heterogeneity in covariance matrices. Consistent with established guidelines [56], the null hypothesis of equal covariance matrices was therefore retained.

**Table 2.** Descriptive statistics of dependent variables across groups.

Construct (n)		Mean (SD)	
Actual behavior (AB)			
Control (30)		3.93 (1.799)	
Experiment (32)		2.75 (2.016)	
Knowledge (K)			
Control (30)		1.82 (0.412)	
Experiment (32)		1.81 (0.494)	
Attitude (A)			
Control (30)		1.79 (0.338)	
Experiment (32)		1.62 (0.541)	
Intended behavior (IB)	
Control (30)		1.92 (0.371)	
Experiment (32)		1.73 (0.463)	
Perceived vulnerability (PV)
Control (30)		2.09 (0.711)	
Experiment (32)		1.85 (0.871)	
Perceived severity (PS)			
Control (30)		2.07 (0.719)	
Experiment (32)		1.41 (0.534)	
Perceived self-efficacy (SE)	
Control (30)		2.75 (0.65)	
Experiment (32)		2.45 (0.672)	
Perceived response efficacy (RE)
Control (30)		1.4 (0.395)	
Experiment (32)		1.41 (0.534)	
Perceived barrier (PB)	
Control (30)		3.35 (0.842)	
Experiment (32)		3.34 (0.689)	
Perceived cues to action (CA)	
Control (30)		2.98 (0.517)	
Experiment (32)		2.55 (0.787)	
Password			
Control (30)		1.82 (0.482)	
Experiment (32)		1.67 (0.59)	
Incident	
Control (30)		2.39 (0.708)	
Experiment (32)		2.07 (0.766)	
Email
Control (30)		1.6 (0.43)	
Experiment (32)		1.49 (0.523)	
Mobile			
Control (30)		1.65 (0.355)	
Experiment (32)		1.65 (0.471)	

### Reliability, Validity, and Assumption Assessment

Table 3 presents internal consistency estimates for the study constructs. Cronbach α values ranged from 0.256 for CA to 0.792 for incident reporting. While several constructs demonstrated acceptable internal consistency, most notably CA (*α*=0.256), password practices (*α*=0.349), and PBs (*α*=0.476) exhibited poor internal consistency. These variables were therefore retained solely as exploratory indicators and were not used to support confirmatory inference or substantive theoretical claims.

**Table 3.** Reliability statistics.

Number	Construct	Cronbach α	Number of items (n)
1	Knowledge (K)	0.660	13
2	Attitude (A)	0.53	9
3	Behavior (B)	0.648	16
4	Perceived vulnerability (PV)	0.654	3
5	Perceived severity (PS)	0.540	3
6	Perceived self-efficacy (SE)	0.655	6
6	Perceived response efficacy (RE)	0.717	3
8	Perceived barriers (PB)	0.476	4
9	Cues to action (CA)	0.256	2
10	Password	0.349	6
11	Incident	0.792	9
12	Email	0.554	7
13	Mobile	0.65	12

### Phishing Click Behavior With Chi-Square Test

Phishing susceptibility was evaluated using an omnibus chi-square test of independence comparing link-click behavior across 3 groups, including control (n=34), CD-primed (n=32), and neutral nonresponders (n=753). Observed click rates were 65% (22/34) in the control group, 44% (14/32) in the CD-primed group, and 53% (396/753) in the neutral group (Table 4; Figure 4). The corresponding 95% CIs (Wilson method) were 0.48‐0.79, 0.28‐0.61, and 0.49‐0.56, respectively.

**Table 4.** Observed contingency table for phishing click behavior.

Outcome	Control (n=34)	CD-Primed (n=32)	Neutral (n=753)
Clicked (n)	22	14	396
Not clicked (n)	12	18	357
Total (n)	34	32	753

The omnibus chi-square test did not detect a statistically significant association between group membership and click behavior, (*χ*²_2_=3.00; n=819=; *P*=.22). The effect size was small (Cramér V=0.060; approximately 95% CI 0.00‐0.12), indicating that differences in click behavior across groups were modest and consistent with sampling variability.

To contextualize the observed pattern within the randomized subset, exploratory descriptive comparisons were computed between the CD-primed and control groups. The CD-primed group exhibited a lower observed click rate than the control group (44% vs 65%), corresponding to an absolute risk difference of −0.21 (95% CI −0.45 to 0.03), a relative risk of 0.68 (95% CI 0.42‐1.08), and an odds ratio of 0.42 (95% CI 0.16-1.14). These estimates are imprecise due to small group sizes and are reported descriptively without confirmatory inference.

Because the Neutral group was not randomized and consists of nonresponders, comparisons involving the Neutral group are observational and may reflect selection bias or baseline differences rather than intervention effects. Overall, the behavioral results indicate, at most, a directional but statistically nonsignificant association between CD exposure and immediate phishing click behavior under real-world conditions.

A post hoc power assessment based on the observed effect size (W=0.060) indicated limited sensitivity to detect effects of this magnitude (power ≈0.32) and is reported descriptively only.

### Assumption Checks

Normality of the dependent variables was assessed using the Shapiro-Wilk test [57]. As shown in Table 5, several variables deviated from normality, which informed the selection of robust and nonparametric analytical approaches.

**Table 5.** Normality test (Shapiro-Wilk).

Construct	-Shapiro-Wilk statistic, W (df)	*P* value
Actual behavior (AB)	0.627 (62)	<.001
Knowledge (K)	0.969 (62)	.11
Attitude (A)	0.954 (62)	.02
Intended behavior (IB)	0.968 (62)	.11
Perceived vulnerability (PV)	0.917 (62)	<.001
Perceived severity (PS)	0.884 (62)	<.001
Perceived self-efficacy (SE)	0.981 (62)	.43
Perceived response efficacy (RE)	0.814 (62)	<.001
Perceived barriers (PB)	0.966 (62)	.08
Cues to action (CA)	0.828 (62)	<.001
Password	0.951 (62)	.01
Incident	0.960 (62)	.04
Email	0.887 (62)	<.001
Mobile	0.931 (62)	.002

### Exploratory Multivariate Analyses

Subsequently, a one-way MANOVA was performed to examine whether group membership was associated with differences in the combined set of dependent variables, including mobile device and SMS use and incident reporting, as shown in Table 6. Statistical significance was evaluated at an ɑ level of .05.

**Table 6.** Correlation (n=62).

Variable	AB^a^	Knowledge	Attitude	IB^b^	PV^c^	PS^d^	SE^e^	RE^f^	PB^g^	CA^h^	Password	IR	Email use	Mobile
AB	—^i^	–0.371^j^	–0.021	–0.050	0.010	0.227	0.090	–0.090	0.113	0.041	0.041	–0.175	–0.027	–0.208
Knowledge	–0.371^j^	—	0.500^j^	0.515^j^	0.133	–0.159	0.440^j^	0.336^j^	–0.328^j^	0.149	0.390^j^	0.495^j^	0.538^j^	0.683^j^
Attitude	–0.021	0.500^j^	—	0.468^j^	0.261^k^	0.213	0.067	0.257^k^	–0.209	0.074	0.394^j^	0.658^j^	0.402^j^	0.382^j^
IB	–0.050	0.515^j^	0.468^j^	—	0.293^j^	0.297^j^	0.432^j^	0.407^j^	–0.401^j^	0.337^j^	0.642^j^	0.637^j^	0.655^j^	0.479^j^
PV	0.010	0.133	0.261^k^	0.293^j^	—	0.383^j^	0.030	0.319^j^	–0.147	0.126	0.217	0.291^j^	0.076	0.050
PS	0.227	−0.159	0.213	0.297^j^	0.383^j^	—	0.062	0.148	–0.128	0.173	0.243^k^	0.272^k^	0.150	–0.019
SE	0.090	0.440^j^	0.067	0.432^j^	0.030	0.062	—	0.366^j^	–0.323^j^	0.537^j^	0.158	0.156	0.431^j^	0.505^j^
RE	–0.090	0.336^j^	0.257^k^	0.407^j^	0.319^j^	0.148	0.366^j^	—	–0.108	0.146	0.172	0.232^k^	0.356^j^	0.350^j^
PB	0.113	–0.328^j^	–0.209	–0.401^j^	–0.147	–0.128	–0.323^j^	–0.108	—	–0.336^j^	–0.264^k^	–0.330^j^	0.236^k^	0.148
CA	0.041	0.149	0.074	0.337^j^	0.126	0.173	0.537^j^	0.146	–0.336^j^	—	0.133	0.182	0.204	0.362^j^
Password	0.041	0.390^j^	0.394^j^	0.642^j^	0.217	0.243^k^	0.158	0.172	–0.264^k^	0.133	—	0.383^j^	0.353^j^	0.210
IR	–0.175	0.495^j^	0.658^j^	0.637^j^	0.291^j^	0.272^k^	0.156	0.232^k^	–0.330^j^	0.182	0.383^j^	—	0.335^j^	0.133
Email use	–0.027	0.538^j^	0.402^j^	0.655^j^	0.076	0.150	0.431^j^	0.356^j^	0.236^k^	0.204	0.353^j^	0.335^j^	—	0.463^j^
Mobile	–0.208	0.683^j^	0.382^j^	0.479^j^	0.050	–0.019	0.505^j^	0.350^j^	0.148	0.362^j^	0.210	0.133	0.463^j^	—

a AB: actual behavior.

b IB: intended behavior.

c PV: perceived vulnerability.

d PS: perceived severity.

e SE: self-efficacy.

f RE: response efficacy.

g PB: perceived barrier.

h CA: cues to action.

i Not available.

j Correlation is significant at the 0.01 level (2-tailed).

k Correlation is significant at the 0.05 level (2-tailed).

Given the inclusion of constructs with low internal consistency, MANOVA is interpreted strictly as a descriptive screening of group differentiation rather than as inferential evidence of multivariate effects. A multivariate effect was observed in the MANOVA. Pillai’s Trace was used for the omnibus multivariate test. The analysis yielded a Pillai’s Trace of 0.442, *F*_14, 47_=2.660; *P*=.006, indicating overall group differences across the combined set of dependent variables. Given the heterogeneous reliability of the included constructs and the limited survey sample size, this result is interpreted as an omnibus, exploratory indication of group differentiation rather than evidence of specific construct-level effects.

The estimated multivariate effect size suggests that approximately 44.2% of the variance in the linear combination of dependent variables was associated with group membership. Assumptions for follow-up univariate analyses were assessed using Levene test across all 14 dependent variables [58]. Although AB and CA showed statistically significant Levene results (*P*<.05), inspection of group standard deviations (Table 2) revealed no substantial imbalance in variance, supporting the robustness of subsequent ANOVA analyses. The CA exhibited very low internal consistency (Cronbach *α*=0.256) and were therefore retained solely as an exploratory indicator, without supporting confirmatory inference.

Follow-up one-way ANOVA tests indicated statistically significant group differences for AB, PS, and CA (Table 7). Findings for constructs with acceptable reliability are interpreted with greater confidence, whereas results involving CA, PBs, and password practices are treated as exploratory, with emphasis placed on effect sizes rather than *P* values. In particular, although statistical differences were observed for CA, this finding is interpreted cautiously due to very low internal consistency and is reported only as an exploratory pattern rather than a substantive effect.

**Table 7.** ANOVA results.

*Construct*	Sum of Squares	df	Mean Square	F	*P* value	Partial eta squared	Noncentrality parameter	Observed power
Actual behavior (AB)	21.682	1	21.682	5.917	.02	0.090	5.917	0.668
Knowledge (K)	0.003	1	0.003	0.013	.91	0.000	0.013	0.051
Attitude (A)	0.452	1	0.452	2.186	.14	0.035	2.186	0.307
Intended behavior (IB)	0.572	1	0.572	3.223	.08	0.051	3.223	0.423
Perceived vulnerability (PV)	0.853	1	0.853	1.340	.25	0.022	1.340	0.207
Perceived severity (PS)	6.753	1	6.753	17.020	<.001	0.221	17.020	0.982
Perceived self-efficacy (SE)	1.365	1	1.365	3.116	.08	0.049	3.116	0.412
Perceived response efficacy (RE)	0.001	1	0.001	0.003	.96	0.000	0.003	0.050
Perceived barrier (PB)	0.003	1	0.003	0.005	.94	0.000	0.005	0.051
Perceived cues to action (CA)	2.950	1	2.950	6.574	.01	0.099	6.574	0.713
Password	0.325	1	0.325	1.110	.30	0.018	1.110	0.179
Incident	1.652	1	1.652	3.028	.09	0.048	3.028	0.402
Email	0.183	1	0.183	0.792	.38	0.013	0.792	0.141
Mobile	0.000	1	0.000	0.000	.99	0.000	0.000	0.050

Post-hoc power analysis indicated that the survey-based MANOVA and ANOVA models achieved approximately 40% power to detect medium-sized effects, confirming that these analyses were underpowered. Consequently, survey-based multivariate and univariate results are interpreted as exploratory. In contrast, the behavioral click-rate analysis, based on the full sample, was sufficiently powered.

## Discussion

### Principal Findings

Despite substantial investment in technical email filtering and security awareness training, health care staff continue to demonstrate high susceptibility to socially engineered emails under real operational conditions [11]. Evidence from field-based phishing simulations suggests that many existing interventions show limited or inconsistent effectiveness when evaluated using objective behavioral outcomes rather than self-reported intentions [52,53]. This limitation has prompted increasing interest in behavioral and psychologically grounded approaches that aim to influence decision-making at the moment of threat exposure, particularly those capable of producing measurable short-term changes in actual user behavior.

In light of this gap, the present study examined whether a brief CD-based priming intervention, delivered immediately prior to a real-world phishing simulation, was associated with differences in phishing-related outcomes among health care staff. The analytical framework comprised one confirmatory behavioral hypothesis and 2 exploratory research questions. The primary and confirmatory hypothesis (hypothesis 1) evaluated whether exposure to the CD prompt was associated with reduced observed phishing susceptibility, operationalized as objective link-click behavior during an in-the-wild phishing simulation. This behavioral outcome served as the study’s primary endpoint and was assessed using an omnibus analysis across all staff. Two exploratory research questions (RQ1 and RQ2) examined whether CD exposure was associated with directional differences in theory-driven security perception constructs derived from the HBM and PMT, as well as with self-reported security practices related to password management, incident reporting, email handling, and mobile-device security.

Given the limited sample size and variable construct reliability, the findings for RQ1 and RQ2 are interpreted as exploratory and hypothesis-generating rather than confirmatory. Accordingly, the discussion first addresses the confirmatory behavioral findings (hypothesis 1), followed by a cautious interpretation of exploratory construct-level and self-reported outcomes.

### Primary Study Finding (Hypothesis 1): Confirmatory Behavioral Outcome

The primary outcome of this study was objective phishing click behavior observed during an in-the-wild simulation [29,59,60]. Although the CD-primed group exhibited a lower observed click-through rate than the control group (44% vs 65%), the prespecified omnibus chi-square test did not detect a statistically significant association between group membership and click behavior. The estimated effect size was small, and CIs were wide, reflecting limited precision due to the modest size of the randomized experimental groups.

Accordingly, the findings do not provide confirmatory statistical support for hypothesis 1. Rather, they indicate a directional, but statistically nonsignificant, association between brief CD-based priming and immediate phishing-click behavior under real-world conditions. This cautious interpretation is warranted because only a subset of participants was randomized to receive the CD prompt, while the neutral group comprised nonresponders and was not assigned to any experimental condition. As such, causal inference is limited to comparisons within the randomized subset, whereas comparisons involving the neutral group are observational and may reflect baseline or selection differences.

When situated within the broader cybersecurity and health informatics literature, the modest magnitude of the observed effect is consistent with prior studies examining behavioral phishing outcomes, which frequently report small and heterogeneous effects following brief or one-time interventions (eg, [61-63]). These findings contrast with studies reporting stronger effects from sustained educational or motivational interventions, which typically target knowledge, risk appraisal, or SE rather than rapid, heuristic-driven decision-making.

This distinction highlights the inherent difficulty of influencing immediate phishing behavior using brief psychological cues alone. From an applied perspective, the present results suggest that CD-based prompts may function as a situational nudge, introducing momentary cognitive friction prior to exposure, rather than as a standalone behavioral control. Their potential value, therefore, lies in complementing existing phishing simulations, awareness training, and technical safeguards within a layered defense strategy.

A notable pattern in the findings is the disconnect between observed phishing behavior and self-reported security perceptions. Although phishing susceptibility was measured using an objective behavioral outcome (link-clicking), effect estimates were imprecise because the randomized comparison groups were small. At the same time, several survey-based constructs showed low internal consistency, limiting confidence in construct-level interpretation. Together, these results are consistent with prior evidence that self-reported security perceptions and intentions do not reliably predict real-world phishing behavior, particularly in high-pressure health care settings.

### Construct-Level Findings With Limited Power: Exploratory Evidence (RQ1-RQ2)

In contrast to the confirmatory behavioral outcome (hypothesis 1), analyses addressing the exploratory research questions (RQ1-RQ2) focused on self-reported psychological perceptions and security practices and are interpreted as hypothesis-generating rather than confirmatory. Although the multivariate analysis indicated an overall group effect (Pillai Trace=0.442; *F*_14,47_=2.66; *P*=.006), this result was derived from a substantially smaller survey subsample (control n=30; CD-primed n=32) and involved multiple dependent variables, thereby limiting statistical sensitivity for reliable construct-level inference.

Follow-up univariate analyses revealed nonsignificant or marginal effects for most perception and practice constructs, including PV, SE, RE, incident-reporting intentions, password management, email handling, mobile device security practices, and self-reported knowledge, attitudes, and intended behavior (*P*=.08-.30). Several constructs, most notably CA, PBs, and password practices, also exhibited low internal consistency, further constraining interpretability and precluding confirmatory conclusions.

While some variables displayed directional or near-significant trends, these patterns do not provide evidence of causal mechanisms nor establish that the CD prompt directly influenced underlying psychological processes [64,65]. Rather, the observed divergence between modest behavioral effects and weak or inconsistent construct-level changes is consistent with prior health care and organizational security research documenting limited correspondence between self-reported perceptions or intentions and objectively observed security behavior [10,18]. Accordingly, the construct-level findings are best viewed as exploratory signals that motivate future research by using larger and more balanced samples, improved psychometric validation, and analytical designs capable of formally testing mediation or mechanism-based pathways.

### Contextual Factors, Baseline Susceptibility, Implications of the Study

Across all study groups, phishing susceptibility remained high, with observed click rates exceeding 50%. This pattern reinforces prior evidence that health care organizations are particularly vulnerable to social engineering attacks, even in settings with established technical defenses, policies, and awareness programs [6,66]. The phishing message deployed in this study leveraged a contemporaneous geopolitical crisis, a strategy commonly observed in real-world phishing campaigns to heighten emotional salience, urgency, and perceived legitimacy [55]. While this design choice enhances ecological validity, it also underscores the context-dependent nature of phishing susceptibility and limits direct generalization beyond comparable threat scenarios.

From an applied perspective, the findings suggest that brief, lightweight psychological prompts may complement existing security awareness and simulation programs by influencing immediate decision-making at the point of exposure. Importantly, the evidence supports this implication only for short-term, observed behavioral responses. The present study does not establish the durability of the observed effects, their transferability across organizational contexts or attack types, or the specific psychological mechanisms through which CD prompting may operate. These limitations highlight the need for longitudinal and replication studies before such interventions can be considered as standalone or broadly generalizable mitigation strategies.

### Conclusion

This study examined whether a brief CD-based priming intervention, delivered immediately prior to a real-world phishing simulation, was associated with differences in phishing susceptibility among health care staff. The primary behavioral analysis indicated a directional but statistically nonsignificant association, with lower observed click rates in the CD-primed group; however, effect estimates were small and imprecise due to limited randomized group sizes. Accordingly, causal inference is restricted to the randomized comparison, while differences involving the neutral group are observational.

Survey-based analyses of security perceptions and self-reported practices were conducted on a smaller subsample, and several constructs exhibited low internal consistency. These findings are therefore considered exploratory and do not support conclusions about psychological mechanisms. Overall, the results suggest that CD-based prompts may function as a lightweight, short-term behavioral nudge under real-world conditions but do not establish a reliable effect.

Larger, fully randomized, and longitudinal studies with improved psychometric validation are needed before such interventions can be considered reliable complements to established cybersecurity controls.

## Supplementary material

10.2196/68051 Multimedia Appendix 1 Questionnaire.

10.2196/68051 Multimedia Appendix 2 Cognitive dissonance message.

10.2196/68051 Checklist 1 CONSORT (Consolidated Standards of Reporting Trials) checklist.

10.2196/68051 Checklist 2 STROBE (Strengthening the Reporting of Observational Studies in Epidemiology) checklist.

## Abbreviations

AB: actual behavior
CA: cues to action
CD: cognitive dissonance
CONSORT: Consolidated Standards of Reporting Trials
HBM: Health Belief Model
KAB: knowledge, attitude, and behavior
MANOVA: multivariate analysis of variance
NSD: Norwegian Center for Research Data
PB: perceived barrier
PMT: Protection Motivation Theory
PS: perceived severity
PV: perceived vulnerability
RE: response efficacy
REK: Regional Committees for Medical and Health Research Ethics of Norway
RQ: research question
SE: self-efficacy
STROBE: Strengthening the Reporting of Observational Studies in Epidemiology
