# Supplementary material for: Cognitive Dissonance–Based Priming Intervention: Randomized Encouragement With in-the-Wild Phishing Simulation Attack in Health Care
Source: J Med Internet Res. 2026 Jun 1;28:e68051. doi: 10.2196/68051 (PMC13225503; doi:10.2196/68051)
Supplement: Multimedia Appendix 1 [file jmir-v28-e68051-s001.docx]

## Questionnaire

Table S1: Nature of questionnaire

| # Item | Construct |
| --- | --- |
| 1. I know that my password and username can be stolen by phishing attacks 2. I think it is unnecessary to use two or more proofs (multi-factor authentication) | KAB with password management [44] |
| such as passwords and codes on SMS to gain access to a website.   1. I prefer NOT to use two or more proofs (multi-factor authentication) such as passwords and code on SMS to access a website. 2. I think it is necessary to report suspicious e-mails or SMS 3. I do NOT report suspicious emails or SMS 4. I know that I should not ignore my colleagues’ poor information security prac- | KAB with incident reporting [44] |
| tices at, for example, nursing home   1. I know that it is not good information security practice to click on a link in an email from an unknown sender 2. I know I can download malicious email attachments, especially from unknown senders 3. I don’t think there is anything wrong with downloading an email attachment | KAB with email use [44] |
| without checking the sender   1. I always check the source or sender before I download an email attachment 2. It is important to pay close attention to phishing attempts 3. I do NOT pay close attention to phishing attempts 4. I have the skills to identify malicious or suspicious links on the hospital mobile phone 5. It is not a bad idea to send sensitive information via voice or SMS | KAB with mobile phone use [44] |
| 1. I may sometimes send sensitive information via voice or SMS 2. I can identify malicious phones in the hospital 3. I feel that the chance of receiving an email with a virus attached is high. 4. I feel that my chance of receiving malware via social media is high. | Perceived vulnerability [44] |
| 1. I believe that my efforts to protect the organization’s information will reduce illegal access. 2. Loss of data as a result of hacking is a serious problem for me. 3. If someone gets access to confidential information about me without my consent | Perceived severity [44] |
| or that I know it is a serious problem for me.   1. If my PC is infected by a virus as a result of a suspicious e-mail attachment being opened, this is a serious problem for me. 2. I know how to identify phishing emails 3. I can create strong passwords on my IT system | Self efficacy [44] |
| 1. I can reveal voice-based phishing attempts 2. Compliance with the information security practices in my organization helps to minimize security breaches | Perceived barrier |
| 1. If I comply with good information security practices, the chance of an information security breach/attack will be reduced 2. Good information security practices help to avoid security breaches. 3. I have been offered training/courses in information security over the past two years | Cuest to action |

Reference:

44. Log in. Nettskjema. URL: <https://nettskjema.no> [Accessed 2025-12-12]
