# Supplementary material for: Cognitive Dissonance–Based Priming Intervention: Randomized Encouragement With in-the-Wild Phishing Simulation Attack in Health Care
Source: J Med Internet Res. 2026 Jun 1;28:e68051. doi: 10.2196/68051 (PMC13225503; doi:10.2196/68051)
Supplement: Multimedia Appendix 2 [file jmir-v28-e68051-s002.docx]

## Appendix B: Cognitive dissonance message

Phishing attacks are a method that cybercriminals use to trick innocent users into clicking on links with the aim of stealing sensitive information or breaching privacy. Through phishing, cybercriminals can block access to health data or the entire network and demand a ransom to unlock access. This is known as ransomware. The integrity of the patient’s information can be deleted or changed, so one cannot trust that the information is correct when providing healthcare. Cyber-attacks can lead to a loss of trust from patients, large fines from regulatory authorities, and, in the worst-case scenario, can lead to the loss of patients’ lives. Based on this information, all employees in the health sector have to follow good routines within cybersecurity to ensure that their actions and attitudes are free of risk. Although most people have good security awareness, sometimes they decide to break security requirements, where they have various excuses that justify the actions, such as “nothing bad can happen”, “no one will know or see it”, “it is a one-off”, “this is not malicious”. These are examples of behavior that contribute to increasing the number of cyberattacks in the healthcare sector.
