# Supplementary material for: Cognitive Dissonance–Based Priming Intervention: Randomized Encouragement With in-the-Wild Phishing Simulation Attack in Health Care
Source: J Med Internet Res. 2026 Jun 1;28:e68051. doi: 10.2196/68051 (PMC13225503; doi:10.2196/68051)
Supplement: Checklist 1 [file jmir-v28-e68051-s003.pdf]

CONSORT 2025 checklist of information to include when reporting a randomised trial*

Section / Topic	No	CONSORT 2025 checklist item description	Reported on page no.
**Title and abstract**			
Title and structured abstract	1a	Identification as a randomised trial	p. 1 (Title & Abstract)
	1b	Structured summary of the trial design, methods, results, and conclusions	p. 1 (Abstract)
**Open science**			
Trial registration	2	Name of trial registry, identifying number (with URL) and date of registration	p. 14 (Methods: Ethics and registration)
Protocol and statistical analysis plan	3	Where the trial protocol and statistical analysis plan can be accessed	pp. 7–10 (Methods: Study design & statistical plan)
Data sharing	4	Where and how the individual de-identified participant data (including data dictionary), statistical code and any other materials can be accessed	p. 28 (Data sharing statement)
Funding and conflicts of interest	5a	Sources of funding and other support (e.g., supply of drugs), and role of funders in the design, conduct, analysis and reporting of the trial	p. 28 (Funding statement)
	5b	Financial and other conflicts of interest of the manuscript authors	p. 28 (Conflicts of interest)
**Introduction**			
Background and rationale	6	Scientific background and rationale	pp. 2–4 (Introduction)
Objectives	7	Specific objectives related to benefits and harms	pp. 4–5 (Objectives and hypotheses)
**Methods**			

Patient and public involvement	8	Details of patient or public involvement in the design, conduct and reporting of the trial	pp. 13–14 (Ethical, privacy, and security considerations)
Trial design	9	Description of trial design including type of trial (e.g., parallel group, crossover), allocation ratio, and framework (e.g., superiority, equivalence, non-inferiority, exploratory)	pp. 6–7 (Study design)
Changes to trial protocol	10	Important changes to the trial after it commenced including any outcomes or analyses that were not prespecified, with reason	pp. 6–7 (Protocol changes / randomized encouragement)
Trial setting	11	Settings (e.g., community, hospital) and locations (e.g., countries, sites) where the trial was conducted	p. 6 (Trial setting)
Eligibility criteria	12a	Eligibility criteria for participants	p. 6 (Eligibility criteria)
	12b	If applicable, eligibility criteria for sites and for individuals delivering the interventions (e.g., surgeons, physiotherapists)	p. 6 (Sites and providers)
Intervention and comparator	13	Intervention and comparator with sufficient details to allow replication. If relevant, where additional materials describing the intervention and comparator (e.g., intervention manual) can be accessed	pp. 4–5; Appendix B p. 31 (Intervention details)
Outcomes	14	Pre-specified primary and secondary outcomes, including the specific measurement variable (e.g., systolic blood pressure), analysis metric (e.g., change from baseline, final value, time to event), method of aggregation (e.g., median, proportion), and time point for each outcome	pp. 8–9 (Outcomes)
Harms	15	How harms were defined and assessed (e.g., systematically, non-systematically)	pp. 13–14 (Harms and ethics)
Sample size	16a	How sample size was determined, including all assumptions supporting the sample size calculation	pp. 9–10 (Sample size and power)
	16b	Explanation of any interim analyses and stopping guidelines	p. 10 (No interim analyses)
Randomisation:			

Sequence generation	17a	Who generated the random allocation sequence and the method used	p. 6 (Sequence generation)
	17b	Type of randomisation and details of any restriction (e.g., stratification, blocking and block size)	pp. 6–7 (Randomization type)
Allocation concealment mechanism	18	Mechanism used to implement the random allocation sequence (e.g., central computer/telephone; sequentially numbered, opaque, sealed containers), describing any steps to conceal the sequence until interventions were assigned	p. 6 (Allocation concealment)
Implementation	19	Whether the personnel who enrolled and those who assigned participants to the interventions had access to the random allocation sequence	pp. 6–7 (Implementation)
Blinding	20a	Who was blinded after assignment to interventions (e.g., participants, care providers, outcome assessors, data analysts)	p. 7 (Blinding not feasible)
	20b	If blinded, how blinding was achieved and description of the similarity of interventions	p. 7 (Blinding not applicable)
Statistical methods	21a	Statistical methods used to compare groups for primary and secondary outcomes, including harms	pp. 8–10 (Statistical methods)
	21b	Definition of who is included in each analysis (e.g., all randomised participants), and in which group	pp. 8–9 (Analysis populations)
	21c	How missing data were handled in the analysis	p. 8 (Missing data handling)
	21d	Methods for any additional analyses (e.g., subgroup and sensitivity analyses), distinguishing prespecified from post-hoc	pp. 9–10 (Exploratory analyses)
**Results**			
Participant flow, including flow diagram	22a	For each group, the numbers of participants who were randomly assigned, received intended intervention, and were analysed for the primary outcome	p. 15; Appendix C p. 33 (Participant flow)
	22b	For each group, losses and exclusions after randomisation, together with reasons	p. 15; Appendix C p. 33 (Losses and exclusions)

Recruitment	23a	Dates defining the periods of recruitment and follow-up for outcomes of benefits and harms	p. 6 (Recruitment dates)
	23b	If relevant, why the trial ended or was stopped	p. 6 (Trial completion)
Intervention and comparator delivery	24a	Intervention and comparator as they were actually administered (e.g., where appropriate, who delivered the intervention/comparator, how participants adhered, whether they were delivered as intended [fidelity])	pp. 15–16 (Intervention delivery)
	24b	Concomitant care received during the trial for each group	p. 6 (Concomitant care)
Baseline data	25	A table showing baseline demographic and clinical characteristics for each group	pp. 16–17 (Baseline characteristics)
Numbers analysed, outcomes and estimation	26	For each primary and secondary outcome, by group:<br>● the number of participants included in the analysis<br>● the number of participants with available data at the outcome time point<br>● result for each group, and the estimated effect size and its precision (such as 95% confidence interval)<br>● for binary outcomes, presentation of both absolute and relative effect size	pp. 18–20 (Outcomes and estimation)
Harms	27	All harms or unintended events in each group	p. 20 (Harms)
Ancillary analyses	28	Any other analyses performed, including subgroup and sensitivity analyses, distinguishing pre-specified from post-hoc	pp. 20–23 (Ancillary / exploratory analyses)
**Discussion**			
Interpretation	29	Interpretation consistent with results, balancing benefits and harms, and considering other relevant evidence	pp. 23–26 (Interpretation)
Limitations	30	Trial limitations, addressing sources of potential bias, imprecision, generalisability, and, if relevant, multiplicity of analyses	pp. 25–26 (Limitations)
