# Supplementary material for: Cognitive Dissonance–Based Priming Intervention: Randomized Encouragement With in-the-Wild Phishing Simulation Attack in Health Care
Source: J Med Internet Res. 2026 Jun 1;28:e68051. doi: 10.2196/68051 (PMC13225503; doi:10.2196/68051)
Supplement: Checklist 2 [file jmir-v28-e68051-s004.pdf]

STROBE Checklist (Combined) – Completed for Neutral Group Analysis

Item	Recommendation	Page No.	Relevant text from manuscript
1a	Study design indicated in title/abstract	p. 1	The study is identified as a randomized encouragement with an in-the-wild phishing simulation in healthcare in the title and abstract.
1b	Balanced abstract summary	p. 1	The abstract provides a balanced summary of background, objectives, methods, results, and conclusions.
2	Scientific background and rationale	pp. 2–4	The introduction outlines the healthcare phishing problem, prior behavioral research, and the rationale for a cognitive dissonance intervention.
3	Objectives and hypotheses	pp. 4–5	The primary hypothesis and exploratory research questions are explicitly stated.
4	Key elements of study design	pp. 6–7	A two-stage hybrid randomized encouragement design with an embedded observational component is described.
5	Setting and dates	p. 6	The study was conducted at a large Norwegian hospital with dates of recruitment and data collection specified.
6a	Eligibility and selection	p. 6	Eligibility included all healthcare staff with valid institutional email addresses; nonresponse was treated as nonreceipt.
7	Variables	pp. 8–9	Variables included objective phishing click behavior and exploratory psychological and self-reported security measures.
8	Data sources and measurement	pp. 8–9	Data sources included survey responses and system-recorded phishing click logs from the simulation platform.
9	Bias	pp. 25–26	Potential selection bias due to voluntary participation and nonrandomized neutral group comparisons is discussed.
10	Study size	p. 9	Study size was constrained by voluntary survey uptake; implications for power are discussed.

11	Quantitative variables	pp. 8–9	Quantitative variables were analyzed using chi-square tests, MANOVA, and descriptive statistics as appropriate.
12a	Statistical methods	pp. 8–10	Statistical methods included a pre-specified omnibus chi-square test and exploratory multivariate analyses.
12c	Missing data	p. 8	Missing survey data were handled through listwise deletion following attention-check screening.
13a	Participants at each stage	p. 15; App. C p. 33	Participant flow is reported for both the randomized encouragement group and the neutral observational group.
13b	Reasons for non-participation	p. 15	Reasons for non-participation include nonresponse to the initial questionnaire invitation.
14a	Descriptive data	pp. 16–17	Demographic and baseline characteristics of survey respondents are presented.
15	Outcome data	pp. 18–20	Observed phishing click outcomes are reported for all three groups.
16a	Main results	pp. 18–20	Main results include click rates, effect sizes, and confidence intervals.
17	Other analyses	pp. 20–23	Exploratory subgroup and multivariate analyses are reported descriptively.
18	Key results	pp. 23–24	Key results are summarized with emphasis on the confirmatory behavioral outcome.
19	Limitations	pp. 25–26	Limitations related to sample size, construct reliability, and causal inference are discussed.
20	Interpretation	pp. 23–26	Findings are interpreted cautiously in light of study design and prior literature.
21	Generalisability	pp. 25–26	Generalizability to other healthcare settings and phishing contexts is addressed.
22	Funding	p. 28	The study reports no external funding and no conflicts of interest.
